# The Association of Self-Reported Birthweight with Lung Function and Respiratory Diseases: Results from a Multi-Centre, Multi-Case Control Study in Italy

**DOI:** 10.3390/ijerph192215062

**Published:** 2022-11-16

**Authors:** Ilaria Tocco Tussardi, Ahmad Tfaily, Francesca Locatelli, Leonardo Antonicelli, Salvatore Battaglia, Roberto Bono, Angelo G. Corsico, Nicola Murgia, Pietro Pirina, Marcello Ferrari, Stefano Tardivo, Deborah L. Jarvis, Giuseppe Verlato

**Affiliations:** 1 Department of Diagnostics and Public Health, Section of Hygiene, University of Verona, 37134 Verona, Italy; 2 Department of Diagnostics and Public Health, Section of Epidemiology and Medical Statistics, University of Verona, 37134 Verona, Italy; 3 Department of Internal Medicine, University Hospital of Ancona, 60131 Ancona, Italy; 4 ‘ProMISE’ (Health Promotion, Mother and Child Care, Internal Medicine and Medical Specialties) Department, University of Palermo, 90133 Palermo, Italy; 5 Department of Public Health and Paediatrics, University of Torino, 10124 Torino, Italy; 6 Department of Internal Medicine and Medical Therapy, University of Pavia, 27100 Pavia, Italy; 7 Pneumology Unit, Foundation I.R.C.C.S. Policlinico San Matteo, 27100 Pavia, Italy; 8 Section of Occupational Medicine, Respiratory Diseases and Toxicology, University of Perugia, 06123 Perugia, Italy; 9 Department of Clinical, Surgical and Experimental Sciences, University of Sassari, 07100 Sassari, Italy; 10 Department of Respiratory Medicine, University of Verona, 37129 Verona, Italy; 11 National Heart and Lung Institute, Section of Genomic and Environmental Medicine, Imperial College London, London SW7 2BX, UK

**Keywords:** adult health, birthweight, early life, lung volumes, respiratory diseases, respiratory health

## Abstract

Early life conditions are associated with lung function and the development of respiratory and non-respiratory illnesses. The relationship with birthweight (BW), however, is conflicting. We examined associations of self-reported BW with lung function and the development of respiratory and also non-respiratory diseases within the GEIRD (Gene–Environment Interaction in Respiratory Diseases) project, an Italian multi-centre, multi-case control study involving cases of COPD, asthma, allergic rhinitis and controls. Multinomial logistic regression was performed with case/control status as response variable; BW as main determinant; and adjusting for sex, age and smoking status. Of the 2287 participants reporting BW, 6.4% (n = 147) had low BW (<2500 g), and this proportion was greater in women than men (7.8% vs. 5.1%; *p* = 0.006). Both men and women with low BW were shorter than those with normal BW (mean ± SD: 160.2 ± 5.5 vs. 162.6 ± 6.5 cm in women, *p* = 0.009; 172.4 ± 6.1 vs. 174.8 ± 7.2 cm in men, *p* < 0.001). Although FEV1 and FVC were reduced in individuals with low BW, this was explained by associations with sex and height. In multivariable analysis, BW was not associated with respiratory diseases in adulthood. However, those with low BW had a higher risk of self-reported hospitalisation for lung disease before the age of two (10.3% vs. 4.1%; *p* < 0.001), severe respiratory infection before the age of five (16.9% vs. 8.8%; *p* = 0.001) and hypertension in adulthood (29.9% vs. 23.7%; *p* = 0.001); however, they had a lower risk of arrhythmia (2.7% vs. 5.8%; *p* = 0.027).

## 1. Introduction

Lung function and the development of respiratory diseases in childhood and adulthood are influenced by intrauterine development [1]. The first studies examining the relationships of birthweight (BW), a marker of intrauterine growth, took place over three decades ago and linked BW with low lung function and mortality from chronic obstructive pulmonary disease (COPD) in older men [2,3].

A recent meta-analysis of studies of adults published up to 2015 [4] found evidence of an association of BW with forced vital capacity (FVC) as assessed by spirometry, but the association with airway obstruction (forced expiratory volume in one second/forced vital capacity—FEV1/FVC) was conflicting, appearing to depend on whether BW was considered as a continuous measure or as low or “normal” BW.

Since that systematic review, it has been reported that low BW is associated with an increased risk of impaired FEV1, FEV1/FVC or both within a large cohort taking part in the UK Biobank [5]. Within the Tucson Children’s Respiratory Study cohort, the risk of a restrictive spirometric pattern (defined as FEV1/FVC greater than the 10th percentile and FVC below the 10th percentile) in adult life decreased with increasing BW, with no association observed for airway obstruction [6].

Some studies have looked at these associations in paediatric and adolescent populations in the Netherlands [7], the UK [8,9,10], Germany [11], Tunisia [12] and China [13], and again the results are varied. Greater weight at birth was associated with higher FEV1 and FVC in a Dutch cohort of 10-year-olds [7] and, in addition, with airway obstruction in children aged 5–11 from England and Scotland [8]. However, no associations were seen in a case–control study of UK adolescents [9], in a German cohort of 15-year-olds [11], in Tunisian children aged 6–16 [12] and in children aged 9–11 years from Guangzhou, China [13]. Within the Avon Longitudinal Study of Parents and Children, FEV1, FVC and forced mid-expiratory flow between 25% and 75% of FVC were associated with BW ages of 8–9 years but were not seen in children of 14–17 years of age [10].

BW has not only been associated with lung function but also with respiratory symptoms and diagnosed respiratory disease. One of the earlier studies showed an increased risk of asthma in Israeli 17-year-olds with lower BW [14], whereas the association was not seen in a Dutch cohort of 10-year-olds [7] and a cohort of 1037 New Zealand children reviewed at 18 years [15]. Neither lung function nor bronchial hyper-responsiveness, an objective marker for asthma, was associated with BW in 11-year-old Norwegian children [16]. When considering adults, reports of strong associations [17,18] again coexist with studies, finding no evidence of an impact of BW on asthma [19,20].

Low BW has been associated with other health conditions, including an increased risk for cardiovascular diseases and events [21,22] and diabetes (especially type 2) [23], while the association with cancer is less clear [24] and controversy remains.

The aim of this study was to investigate the associations between BW and lung function, respiratory and non-respiratory diseases from over 2000 adults living in Italy.

## 2. Materials and Methods

### 2.1. Study Population: The GEIRD Project

We used information collected within the GEIRD (Gene–Environment Interaction in Respiratory Diseases) project, the details of which have been described elsewhere [25]. This multi-centre, multi-case control study in the general population enrolled 2720 participants aged 20–87 years between 2008 and 2014, at seven assessment centres throughout Italy. In four of these centres, only people aged <65 years were sampled, and in one centre only people aged ≥65. GEIRD sent a standardized screening questionnaire (Stage 1) to general population samples asking about symptoms of asthma, allergic rhinitis and COPD/chronic bronchitis (CB). According to the responses, subjects were classified as probable cases of asthma, allergic rhinitis, COPD/CB or probable controls. All probable cases of asthma and COPD/CB, along with a 60% random sample of the probable cases of allergic rhinitis and a 30% random sample of probable controls, were referred to a clinical centre where they underwent further assessment (Stage 2). This included an administered questionnaire; lung function tests (slow and forced spirometry as described in the study protocol available at: http://biometria.univr.it/sito_GEIRD/measurement_protocols.html (accessed on 9 October 2022) [26] and consistent with the ATS/ERS guidelines [27]); a bronchodilator reversibility test; bronchial reactivity testing with methacholine; venesection for genotyping and specific IgE assay; height (cm), weight (kg) and blood pressure measurements; and skin prick tests for common allergens [26]. Cases were defined as follows:

- COPD: post-bronchodilator FEV1/FVC <70%.
- Current asthma: ever presenting asthma and asthmatic symptoms or the use of asthma medication in the last year OR a positive methacholine challenge test (provocation dose 20 <4 mg) OR evidence of reversible airway obstruction (pre-bronchodilator FEV1/FVC <70% plus positive reversibility test) with a history of asthma (irrespective of symptoms/medication usage). Positive response to a bronchodilator defined as an increase of ≥12% and ≥200 mL as an absolute value compared with baseline in FEV1.
- CB: not a case of COPD or asthma but with chronic (>3 months/year for at least two years) cough or phlegm.
- Allergic rhinitis: nasal allergies or nasal problems in the presence of animal, pollens and dust, plus a positive skin prick test.
- Control: no nasal/respiratory symptoms and/or conditions reported in the questionnaire or at clinical assessment.

Participants were asked their weight at birth. Those who were not able to provide their BW in grams were asked whether they had been “born underweight (weight at birth under 2500 g)”. To distinguish between effects on restrictive and on obstructive patterns of lung function, FEV1, FVC and FEV1/FVC measures were considered for the present study.

### 2.2. Statistical Analysis

The main determinant in the present study was self-reported BW, which was considered both as a dichotomous variable (<2500 vs. ≥2500 g) and as a continuous variable (in secondary analysis, which was limited to the subgroup of participants who provided actual BW). Reported BW <500 and >6000 g were considered unreliable and were excluded from the analysis.

The main outcomes were case/control status (COPD/asthma/allergic rhinitis/CB/control), measures of lung function (FEV1 and FVC % predicted, FEV1 and FVC as absolute volumes and FEV1/FVC—Tiffenau index [26,28]) and anthropometric measurements (height, weight, body mass index—BMI) in adulthood. Additional outcomes were self-reported respiratory events and conditions in childhood (hospitalisation for lung disease before 2 years of age and severe respiratory infection before 5 years) and non-respiratory diseases in adulthood (cardiovascular diseases, diabetes type 1 or 2 and the diagnosis of cancer).

Most quantitative outcomes (weight at birth, weight and BMI in adulthood, FEV1, FVC and FEV1/FVC) were non-normally distributed in several subgroups. As a consequence, non-parametric tests were used: the Mann–Whitney U test for the comparison of two groups and the Kruskal–Wallis test for the comparison of more than two groups.

The lung function measured was investigated using quantile (median) regression, where lung volume (FEV1, FVC, FEV1 % predicted and FVC % predicted) was the response variable; BW (<2500 or ≥2500 g) was the main determinant; and case/control status, sex, age class (20–34, 35–49, 50–64, 55–87 years), height and smoking status were the potential confounders. Standard errors were adjusted for intra-centre correlation.

For other respiratory outcomes, in accordance with the multi-case control study design, a multinomial logistic regression model was conducted with the response variable as the case/control status (COPD/asthma/allergic rhinitis/control) and the main determinant was self-reported BW (dichotomised as <2500 or ≥2500 g), with sex, age and smoking status (never/past/current smoker) as potential confounders. Results were synthesized through the relative risk ratios (RRRs), adjusting standard errors for intra-centre correlation. As there were only 28 cases of CB without COPD, asthma and allergic rhinitis, and these cases were removed from the multinomial logistic regression.

Multivariable analyses (multinomial logistic regression and quantile regression) were repeated considering BW as a continuous variable.

Statistical analyses were performed using Stata 16 (Stata Corporation, College Station, TX, USA).

## 3. Results

Overall, 2287 participants provided information as to whether they were born underweight or normal weight, and of these, 1665 (72.8%) provided actual BW. Mean age (±SD) of the 2287 was 49 ± 13 years. Out of 2287, 147 participants (6.4%) were classified as low BW.

Out of 2287 participants, the case/control status was available for 2049 individuals, the others having declined full clinical assessment. Of the 2049, 674 (32.9%) were cases of allergic rhinitis, 671 (32.7%) of asthma, 60 (2.9%) of COPD and 21 (1.1%) of CB, while 623 (30.4%) were controls. The prevalence of cases and controls was similar in the smaller group of the 1665 who provided actual BW (1502 with known case/control status). Table 1 shows the prevalence of low BW by sex, age group (corresponding to two subsequent phases of the GEIRD study), age class and centre at interview. Low BW was more commonly reported by women than men (7.8% vs. 5.1%; *p* = 0.006) and by younger than older adults (6.7% vs. 4.6%; *p* = 0.241).

In the 1665 participants reporting actual BW, mean BW (±SD) was 3353 ± 672 g and was lower in females than males (3221 ± 633 g vs. 3504 ± 683 g; *p* < 0.001).

Table 2 shows that both females and males reporting low BW were shorter than those with normal BW (*p* = 0.009 and *p* < 0.001, respectively) but had similar weight and BMI.

As shown in Table 3, FEV1 and FVC % predicted (which accounts for age, sex and height) did not differ significantly in individuals with low compared to normal BW. These differences were significant only when FEV1 and FVC were considered as absolute values. The Tiffenau index expressed as FEV1/FVC was not associated with BW (Table 3).

**Figure 1:**
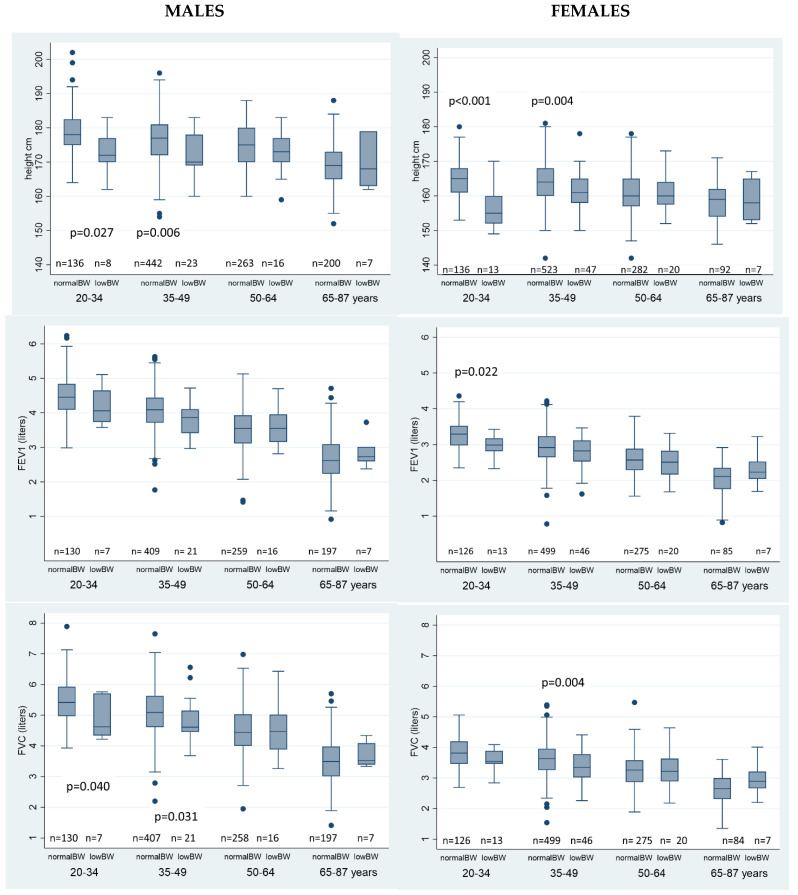
Differences in height, FEV1 and FVC between individuals by birthweight category as a function of sex and age.

Figure 1 shows the association of BW (as dichotomous variable) with outcomes stratified by sex and age. There was a tendency for the differences in height, FEV1 and FVC in those reporting low BW who were born more recently (20–34 and 35–39 years), but again no differences were seen in predicted FEV1 and FVC.

After adjustment for case/control status, there was no difference in median FEV1 and FVC % predicted by BW category (Table 4). Further adjustment for smoking made little difference to these estimates (not shown).

Supplementary Table S1 shows there was also no difference in median FEV1 and FVC absolute values by BW category after adjustment for age, sex, height and case/control status. The difference in median FEV1 by BW category was significant when controlling for case/control status and age (*p* < 0.001) but not when adding to the model of either sex (*p* = 0.110) or height (*p* = 0.608). In the smaller sample with information on actual BW (n = 1665), there was no difference in median FEV1 or FVC with the same adjustments as above. The difference in median FEV1 by a 100 g increase in BW was significant when controlling for case/control status and age (*p* < 0.001) but not when adding sex and height (*p* = 0.490).

Although median BW was lower in participants with COPD, adjusted multinomial analyses showed no association of BW considered as a binary measure with respiratory conditions in adults (Table 5).

Likewise, no association was observed when BW was taken as a continuous variable (RRR of 100 g increase in BW = 1.02, CI 95% 0.98–1.06 for COPD; 0.99, CI 95% 0.96–1.01 for asthma; and 0.98, CI 95% 0.95–1.01 for allergic rhinitis).

Participants reporting low BW were more likely to report events suggestive of poor respiratory health in childhood. Adjusted analyses showed a higher risk of being hospitalised for lung disease before the age of two and of having severe respiratory infections before the age of five (*p* < 0.001 and *p* = 0.001, respectively) (Table 6). Participants reporting low BW were also more likely to report arrhythmia and hypertension in adulthood (*p* = 0.027 and *p* = 0.001, respectively) but not coronary thrombosis, angina, diabetes and cancer.

We saw no evidence of an association of BW considered as a continuous measure with adult conditions.

## 4. Discussion

In this cross-sectional study of over 2000 Italian adults, we did not observe associations between self-reported low BW and lower lung function, namely FEV1 and FVC. This was largely explained by the associations of BW with sex and height. Indeed, in multivariable analysis BW was a significant predictor of lung volumes when controlling for age and case/control status but not when also considering sex and height, both with BW as a dichotomous and as a continuous variable. We found no associations of BW, both as binary and continuous measure, with adult respiratory diseases, but those reporting low BW were more likely to report events suggestive of poor respiratory health in childhood and hypertension in adulthood.

In our population, low BW was more prevalent in females. It is well established in the literature that BW is lower in females than males [29,30,31], and therefore, it is not surprising that in our study women were more likely to report a BW lower than 2500 g. Other reports have also shown that low BW is associated with shorter attained height in adults [21,32,33,34], and in our study, a too low BW was associated with shorter stature.

With regard to lung function, our results were not in line with the recent meta-analysis by Saad et al., which indicated an association of BW with adult FVC [4]. Most of the studies included in the meta-analysis adjusted lung volumes for age, sex and height. However, when looking at the results of the secondary analysis of the association of BW (continuous exposure) with FVC (analysis were repeated after adding the latest follow-up results), the combined estimate for the association was not significant (*p* = 0.48). Additionally, in the analysis of BW as a binary exposure, the standardized mean difference in FVC between normal and low BW was not strongly significant (*p* = 0.048), and after excluding the only study that did not adjust for height, the association was not significant (*p* = 0.71). The association of BW with FEV1/FVC was reported by the authors as much weaker than with FVC, with the inconsistencies of findings across studies, especially in the meta-analysis of BW as a continuous exposure, and this is in line with our results. Our results from subgroup analyses by age were in line with the meta-analysis, showing a stronger association between BW and FVC in younger compared to older adults. In our study, we were able to also observe this association with FEV1, especially in women. These results might be the effect of an increasing impact of environmental factors (e.g., smoking, air pollution, diet) throughout lifetime.

It is of note that the study by Saad et al. [4] did not look at FEV1. A meta-analysis on BW and lung function from 2005 by Lawlor et al. [35] analysed only FEV1 on seven studies pooled with the results from the British Women’s Heart and Health study and reported a positive association between BW and FEV1 adjusted for age, smoking and height. However, the study concluded that BW per se was unlikely to have a direct effect on adult lung function, as they observed a difference of just 0.05 L in FEV1 by 1 kg increase in BW in the pooled analysis. The authors suggested that BW might act as a proxy marker for environmental exposure that influences birth size and ultimately have an effect on adult lung function. Subsequently, a longitudinal birth cohort study from 2006 on 5390 Finns by Canoy et al. [36] found positive associations between BW and both FEV1 and FVC adjusted for sex, height, BMI and other confounders, such as smoking and physical activity. Indeed, our results differ from these observations, as in our population, low BW was associated with reduced lung function, but this reduction was explained by reduced height and female prevalence.

As regards the analyses on BW and respiratory conditions, our results are in agreement with previous studies, showing no association with respiratory outcomes in adulthood, namely asthma [19,20]. With regard to COPD, a systematic review from 2021 by Savran and Ulrik [37] explored early life insults as determinants of COPD and reported only one prospective cohort study on 250,000 Swedes detecting a significant increase in risk of obstructive airway disease with decreasing BW [38]. The study, however, considered low BW as <2000 g and <2100 g for females and males, respectively, and the increase in risk was detected in women but not in men. The relationship between BW and postnatal allergy was explored in a systematic review and meta-analysis from 2019 by Wooldridge et al. [39] and the risk of allergic rhinitis was not associated with BW, which is in line with our results. The meta-analysis on the risk of allergic asthma could not be conducted due to an insufficient number of available studies.

Our results on respiratory health in childhood are in line with studies showing an increased risk of hospitalisation and respiratory problems during childhood for children of low BW [40,41,42,43]. A recent follow-up study on 118,166 German children assessed how the cumulative costs for hospital treatment in the first 4 years of life of low BW infants are about EUR 1300 higher than for infants of normal BW [41].

Despite the limited power, in our study population we detected an increased risk of hypertension in individuals reporting low BW, and this finding is in line with previous studies, supporting the role of prenatal environment in the development of adult chronic and cardiovascular diseases [3,21,22,44]. A systematic review from 2015 by Kelishadi et al. [45] on the association of low BW with cardiovascular diseases reported how 58.5% of risk factors and cardiovascular diseases were statistically significant for the “Barker hypothesis” (adverse influences during intrauterine life could lead to permanent changes in metabolism and physiology, which result in elevated susceptibility to adulthood chronic diseases) [3]. A later meta-analysis by Mohseni et al. [46] indicated a positive association between low BW and total cardiovascular risk. Most recently, a study of 256,787 individuals from the UK Biobank observed the nonlinear inverse associations of BW with the risk of coronary heart disease, stroke and heart failure [47]. Overall, the most recent literature seems to support the Barker hypothesis [3] with reference to cardiovascular risk and diseases. It is of note that in our study population, we detected a decreased risk of arrhythmia in individuals reporting low BW, whereas we would have expected a higher risk. The number of cases was small, however, so it is difficult to compare these results with those of larger studies that have found an association between low BW and an increased cardiovascular risk in adulthood.

Our results on the association of BW with diabetes were not in line with the most recent literature, supporting relationships between BW and diabetes, especially type 2. A meta-analysis from 2018 by Zhao et al. [48] showed a higher risk of diabetes in subjects of low BW. A later dose-response meta-analysis by Tian et al. [49] reported a reduction of risk of 12% per 500 g increase in BW. However, in this second meta-analysis, the authors highlighted a high heterogeneity in the included studies, higher in European than in American and Asian studies and also higher in self-reported diabetes mellitus than from medical records. In our population, it is difficult to make considerations about the relevance of the results anyhow, as individuals with self-reported type 1 and 2 diabetes were analysed together. As regards the association with type 1 diabetes, findings from the literature still appear conflicting, and a recent multi-centre large cross-sectional study on 181,786 Chinese children of 3–18 years of age did not detect a significant association [50].

With regard to the association of BW with cancer risk in adulthood, the literature seems to lean towards a link, but the risk apparently increases for higher BW and only for some cancers (breast, prostate and testicular) [51]. Recently, more attention has been paid to the effect of BW on cancer prognosis. A meta-analysis on the association of BW and cancer mortality by Risnes et al. [44] showed that BW in men was positively associated with cancer mortality, whereas in women there was no strong evidence. Later, a meta-analysis by Sharma et al. [52] found conflicting results, with BW associated with the poorer prognosis of some cancers (prostate) but not others (breast). However, this aspect was not investigated in our study.

Conflicting results in the literature have been attributed to the differences in sample sizes and adjustment for confounding factors that were considered within the studies. One important confounder is gestational age, as prematurity is a strong predictor of BW and has been associated with lower lung function in adulthood [53] and also with asthma symptoms both in children [54] and adults [17]. In a recent study investigating the effect of prematurity on lung function, very-to-moderate prematurity was associated with obstructive lung function deficits, including COPD into the sixth decade [55]. However, disentangling the effects of BW and gestational age is complex, also due to the difficulties in estimating gestational age reliably. Moreover, gestational age is not commonly available from the studies. In the above-cited review by Saad et al. [4] less than half of the included studies provided any information about preterm conditions, limiting the understanding of what could mediate the association of BW with lung function in adult life.

### Limitations

An important limitation for this study was the use of self-reported clinical information. Some studies have stated that self-reported BW correlates well with hospital records [36] and this would seem reasonable especially with regard to the dichotomous information of being born normal weight or underweight but absolute levels of accuracy may not be optimal. The decision to use self-reported information and not official records, is a methodological limitation of this study, which may explain the detection of results that conflict with the current literature.

Additionally, there have been authors warning against the evaluation of BW as a binary exposure, as this might lead to a loss in statistical power [4]. However, in the present study, BW was considered both as binary and continuous exposure.

## 5. Conclusions

In our study population, we were not able to see any association of self-reported low BW with asthma, allergic rhinitis or COPD. The reduced FEV1 and FVC in individuals reporting low BW was mediated by a reduction in height and by a larger proportion of women in the population.

However, we did see that those reporting low BW were also more likely to report more severe respiratory infections and hospitalisation for respiratory conditions during childhood and more hypertension in adulthood. When these findings are considered, it is essential to acknowledge that information on BW was reported by the participants themselves in this study.

## Figures and Tables

**Table 1:** Prevalence of low BW (<2500 g) by sex, age class and centre of interview (n = 2287).

	Low BW, n	Prevalence (%)	*p* Value ^1^
**Sex**			0.006
Females	90/1150	7.8	
Males	57/1137	5.1	
**Age class**			0.750
20–34	24/311	7.7	
35–49	75/1014	7.4	
50–64	35/535	6.5	
≥65	13/280	4.6	
**Centre and age group**			
**Torino**			
20–64	13/245	5.3	
**Pavia**			
20–64	18/230	7.8	
**Verona**			
20–64	82/1162	7.1	
≥65	7/102	6.9	
**Ancona**			
20–64	5/86	5.8	
**Terni**			
20–64	3/76	4.0	
**Sassari**			
20–64	13/208	6.3	
≥65	3/123	2.4	
**Palermo**			
≥65	3/55	5.5	
**Total**			
20–64	134/2007	6.7	0.241
≥65	13/280	4.6	

^1^ Significance tested by the Chi-square test.

**Table 2:** Height, weight and BMI measures by birthweight and sex.

	n	Mean ± SD or Median [p25–p75]	*p* Value ^1^
**Height (cm) ^#^**			
Females			**0.009**
Low BW	87	160.2 ± 5.5	
Normal BW	1033	162.6 ± 6.5	
Males			**<0.001**
Low BW	54	172.4 ± 6.1	
Normal BW	1041	174.8 ± 7.2	
**Weight (kg)**			
Females			0.120
Low BW	87	59 [52–70]	
Normal BW	1031	61 [55–70]	
Males			0.139
Low BW	54	75 [70–83]	
Normal BW	1042	79 [72–86]	
**BMI (kg/m^2^)**			
Females			0.840
Low BW	87	23.0 [20.9–26.4]	
Normal BW	1031	23.2 [20.8–26.3]	
Males			0.754
Low BW	54	25.5 [23.0–27.8]	
Normal BW	1039	25.8 [23.5–28.4]	

^1^ Significance tested by the *t*-test and Mann–Whitney U test. ^#^ High was normally distributed.

**Table 3:** Lung function measures by birthweight.

	n	Median [p25–p75]	*p* Value ^1^
**FEV1 (L)**			0.019
Low BW	137	3.01 [2.60–3.45]	
Normal BW	1980	3.16 [2.65–3.86]	
**FVC (L)**			0.003
Low BW	137	3.68 [3.19–4.34]	
Normal BW	1976	3.91 [3.33–4.81]	
**FEV1 PRED (%)**			0.837
Low BW	136	98.72 [91.14–108.10]	
Normal BW	1970	99.30 [90.42–107.94]	
**FVC PRED (%)**			0.956
Low BW	136	98.73 [88.94–108.41]	
Normal BW	1966	98.90 [90.63–107.06]	
**FEV1/FVC (%)**			0.077
Low BW	137	81.25 [77.75–85.67]	
Normal BW	1976	80.46 [75.94–84.82]	

^1^ Significance tested by the *t*-test and Mann–Whitney U test.

**Table 4:** Differences (95% CI) in median FEV1 % predicted and FVC % predicted by birthweight, estimated by median regression and adjusting standard errors for intra-centre correlation (n = 2049).

	Difference (95% CI) in Median FEV1 % Pred	*p* Value	Difference (95% CI) in Median FVC % Pred	*p* Value
**Low vs. normal BW**	0.40 (−1.49; 2.30)	0.675	−0.82 (−2.94; 1.30)	0.447
**COPD**	−28.31 (−33.81; −22.81)	<0.001	−7.13 (−17.14; 2.87)	0.162
**Asthma**	−6.34 (−7.74;−4.94)	<0.001	−1.32 (−4.21; 1.56)	0.369
**Allergic rhinitis**	−1.80 (−2.88;−0.71)	0.001	−0.004 (−1.91;1.92)	0.996

**Table 5:** Association of case status with birthweight with adjustment for sex, age and smoking status (n = 2049). RRR with 95% CI and *p* values were computed by a multinomial logistic regression, where standard errors were adjusted for intra-centre correlation.

	COPD	Asthma	Allergic Rhinitis
	RRR (CI) *p* Value		
**BW (low vs. normal)**	1.55 (0.75–3.17) 0.235	1.05 (0.66–1.67) 0.841	1.18 (0.88–1.57) 0.272
**Sex (female vs. male)**	0.33 (0.22–0.50) <0.001	1.01 (0.88–1.14) 0.937	1.16 (0.97–1.38) 0.100
**Age (per 10** **-year increase)**	1.60 (1.41–1.81) <0.001	0.72 (0.64–0.81) <0.001	1.01 (0.88–1.14) 0.926
**Smoking habits**			
**Past vs. never smoker**	1.18 (0.73–1.92) 0.498	1.21 (1.08–1.35) 0.001	1.05 (0.96–1.15) 0.297
**Current vs. never smoker**	3.10 (2.13–4.51) <0.001	1.51 (1.25–1.82) <0.001	1.46 (1.32–1.60) <0.001

Base outcome = control.

**Table 6:** Association between birthweight and respiratory conditions in childhood and non-respiratory diseases in adulthood.

	Prevalence in Normal BW, n (%)	Prevalence in Low BW, n (%)	*p* Value ^1^	OR (95% CI)	*p* Value ^2^
**Hospitalisation for lung disease before age 2**	88/2138 (4.1)	15/146 (10.3)	0.003	2.00 (1.41–2.84)	<0.001
**Severe respiratory infection before age 5**	184/2087 (8.8)	23/136 (16.9)	0.003	2.65 (1.47–4.80)	0.001
**Coronary thrombosis**	38/2136 (1.8)	3/147 (2)	0.745	2.40 (0.63–9.09)	0.198
**Angina**	32/2135 (1.5)	1/146 (0.7)	0.720	0.60 (0.08–4.70)	0.624
**Arrhythmia**	124/2134 (5.8)	4/143 (2.7)	0.138	0.54 (0.31–0.93)	0.027
**Stroke ^3^**	29/2138 (1.4)	0	0.255	---	---
**Hypertension**	507/2136 (23.7)	44/147 (29.9)	0.091	1.71 (1.25–2.36)	0.001
**Diabetes (type 1 and 2)**	72/2136 (3.4)	6/147 (4.1)	0.636	1.02 (0.52–1.98)	0.963
**Cancer**	125/2135 (5.8)	6/145 (4.1)	0.465	0.78 (0.44–1.37)	0.386

^1^ Significance tested by Chi-square test. ^2^ Odds ratios and *p* values were computed by a logistic regression model, controlling for sex, age class and case/control status. ^3^ Multivariable analysis was not performed for stroke, as this event was not reported by participants with low BW.

## Data Availability

The datasets used and analysed during the current study are available from the corresponding author upon reasonable request.

## Supplementary Materials

The following supporting information can be downloaded at: https://www.mdpi.com/article/10.3390/ijerph192215062/s1, Table S1: Adjusted^1^ differences (95% CI) in median FEV1 and FVC by birthweight, case status, sex, age and height, estimated by median regression and adjusting standard errors for intra-centre correlation (n = 2049).
