# Supplementary material for: The Association of Self-Reported Birthweight with Lung Function and Respiratory Diseases: Results from a Multi-Centre, Multi-Case Control Study in Italy
Source: Int J Environ Res Public Health. 2022 Nov 16;19(22):15062. doi: 10.3390/ijerph192215062 (PMC9690666; doi:10.3390/ijerph192215062)
Supplement: Supplementary file 1 [file ijerph-19-15062-s001.zip › ijerph-1990472-supplementary.pdf]

Supplementary Materials

Table S1. Adjusted[1] differences (95% CI) in median FEV1 and FVC by birthweight, case status, sex, age and height, estimated by median regression and adjusting standard errors for intra-centre correlation (n = 2049).

	**Difference (95% CI) in Median FEV1 (mL)**	***p* Value**	**Difference (95% CI) in Median FVC (mL)**	***p* Value**
**Low vs. normal BW**	9 (−60;77)	0.806	−10 (−81;63)	0.795
**COPD**	−925 (−1024;−825)	**<0.001**	−333 (−655;−10)	**0.043**
**Asthma**	−230 (−294;−166)	**<0.001**	−52 (−186;83)	0.450
**Allergic rhinitis**	−78 (−118;−38)	**<0.001**	−8 (−117;101)	0.882
**Sex (female vs. male)**	−572 (−643;−501)	**<0.001**	−675 (−758;−592)	**<0.001**
**Age (per class)**				
**35-49**	−270 (−339;−201)	**<0.001**	−184 (−277;−92)	**<0.001**
**50-64**	−611 (−659;−561)	**<0.001**	−493 (−536;−449)	**<0.001**
**≥65**	−1172 (−1520;−825)	**<0.001**	−1093 (−1349;−837)	**<0.001**
**Height (per cm)**	38 (36;42)	**<0.001**	54 (52;58)	**<0.001**

[1] Adjusted for all factors in the table.
